# 单倍体造血干细胞移植模式下供者HLA-Bw4对移植后NK细胞重建及移植相关死亡风险的影响

**DOI:** 10.3760/cma.j.cn.121090-20240314-00094

**Published:** 2024-05

**Authors:** 明 赵, 郑丽 徐, 星星 余, 亦扬 丁, 英军 常, 晓辉 张, 晓军 黄, 翔宇 赵

**Affiliations:** 1 北京大学人民医院、北京大学血液病研究所、国家血液系统疾病临床医学研究中心，北京市造血干细胞移植治疗血液病重点实验室，北京 100044 Peking University People's Hospital, Peking University Institute of Hematology, National Clinical Research Center for Hematologic Diseases, Beijing Key Laboratory of Hematopoietic Stem Cell Transplantation for Hematologic Diseases, Beijing 100044, China; 2 厦门大学第一附属医院血液科，厦门 361003 Department of Hematology, The First Affiliated Hospital of Xiamen University, Xiamen 361003, China

**Keywords:** HLA-Bw4, NK重建, 移植后环磷酰胺, 移植相关死亡率, HLA-Bw4, NK reconstitution, PT-Cy, Transplantation-related mortality

## Abstract

**目的:**

探讨在接受来自母体或旁系供者的非去T细胞单倍体造血干细胞移植时，供者HLA-Bw4表达对NK细胞重建和移植预后的影响。

**方法:**

本研究前瞻性入组了32例接受来自母体或旁系的供者非去T细胞单倍体移植患者（队列1），评估供者HLA-Bw4表达对NK细胞重建的影响；同时回顾性研究了278例接受来自母体或旁系的供者非去T细胞单倍体移植患者（队列2），分析供者HLA-Bw4对HSCT预后的影响。同时还分别比较了在接受移植后环磷酰胺（PT-Cy）和不接受PT-Cy的情况下，供者HLA-Bw4表达对HSCT预后影响的差异。

**结果:**

编码HLA-Bw4的供者促进了NK细胞的重建和功能恢复，PT-Cy体内应用并未影响供者HLA-Bw4促进NK细胞重建。编码HLA-Bw4的供者与移植相关死亡率（Transplantation-related mortality，TRM），尤其是感染相关的TRM（infection-related TRM）降低相关，PT-Cy体内应用并未影响供者HLA-Bw4降低TRM。

**结论:**

在进行来自母体或旁系供者的非体外去T细胞单倍体移植时，编码HLA-Bw4的供者有助于快速NK细胞重建和功能恢复，并且与较低的TRM（尤其是感染相关TRM）显著相关。这些结果表明，供者HLA-Bw4的表达对于单倍体移植患者的临床结果具有重要意义。因此，在供受者选择和移植策略中，考虑供者HLA-Bw4可能具有临床意义。

## Abstract (en)

**Objective:**

To investigate the impact of donor human leukocyte antigen（HLA）-Bw4 expression on natural killer（NK）cell reconstitution and transplant outcomes in recipients undergoing haploidentical hematopoietic stem cell transplantation（HSCT）from maternal or related donors without ex vivo T-cell depletion.

**Methods:**

This study prospectively enrolled 32 patients who received T-replete haploidentical HSCT from maternal or collateral donors（cohort 1）to evaluate the facilitating effect of donor HLA-Bw4 expression on NK cell reconstitution. Furthermore, a retrospective analysis was conducted on 278 patients who underwent T-replete haploidentical HSCT from maternal or collateral donors（cohort 2）to analyze the impact of donor HLA-Bw4 expression on HSCT outcomes. Thus, a comparison was made between the effects of donor HLA-Bw4 expression on HSCT outcomes in patients receiving or not receiving post-transplant cyclophosphamide（PT-Cy）conditioning.

**Results:**

Donors expressing HLA-Bw4 alleles facilitated NK cell reconstitution and functional recovery, which remained unaffected by PT-Cy. Donors with HLA-Bw4 expression were associated with reduced transplant-related mortality（TRM）, particularly mortality related to infections. The use of PT-Cy did not impact the ability of donor HLA-Bw4 to decrease TRM.

**Conclusion:**

In haploidentical HSCT from maternal or related donors without ex vivo T-cell depletion, the presence of donor HLA-Bw4 expression promotes rapid NK cell reconstitution and functional recovery and is significantly associated with lower TRM, especially infection-related mortality. These findings underscore the clinical significance of donor HLA-Bw4 expression in patients who underwent HSCT. Hence, the consideration of donor HLA-Bw4 in recipient selection and HSCT strategies holds important clinical implications.

造血干细胞移植（Hematopoietic stem cell transplantation，HSCT）是治疗血液系统恶性肿瘤的主要选择。移植后，自然杀伤（Natural killer，NK）细胞是恢复速度最快的免疫细胞[1]–[4]。NK细胞的活性受到一系列细胞表面受体介导的抑制和激活信号的调控，其中包括抑制和激活型杀伤细胞免疫球蛋白样受体（Killer cell immunoglobulin-like receptors，KIR）[5]。KIR3DL1与其同源配体HLA-Bw4相互作用，而KIR2DL3与其同源配体HLA-CAsn-80、KIR2DL1与其同源配体HLA-CLys-80相互作用。NK细胞功能的增强是通过KIR识别HLA Ⅰ类分子实现的，这个过程被称为教育或许可[6]–[7]。

在本研究中，我们重点研究了NK细胞的KIR3DL1亚群。先前的研究发现，KIR3DL1及其配体HLA-Bw4的基因组合与HIV感染中进展缓慢的获得性免疫缺陷综合征密切相关[8]。此外，KIR3DL1和HLA-Bw4的相互作用关系的不同亲和力组合还被证明能够预测血液病的临床结果。一项研究表明，具有强抑制性KIR3DL1/HLA-Bw4组合与更高的复发和死亡率相关，而对于从无关供者接受移植的急性髓系白血病（Acute myeloid leukemia，AML）患者而言，具有弱或非抑制性组合则具有保护作用[9]。然而，在另一项关注基于细胞因子的免疫疗法的研究中，Hallner等[10]的研究揭示，携带HLA-B-21M（很少含有Bw4结构域）的受试者，在体内NKG2A^+^ NK细胞功能上表现出较强的能力，显示出更强的抗白血病能力。因此，HLA-B位点与NK细胞功能之间的相互作用对于血液系统恶性肿瘤的临床预后具体影响仍不确定，需要进一步的研究。

在HLA不合的移植环境中，NK细胞的教育过程变得更加复杂，因为移植后供者和受者的HLA会混合存在，这对于NK细胞的教育过程至关重要[11]–[13]。此外，许多药物也会干扰NK细胞的教育过程，进一步增加了供者和宿主之间的HLA和KIR组合在预测方面的复杂性。研究表明，移植后环磷酰胺（Posttransplant cyclophosphamide，PT-Cy）可以消除增殖的供者来源的NK细胞，移植后两周内受者体内会出现再生的NK细胞，这些细胞主要具有未成熟的表型，并在移植后3个月部分恢复[14]–[15]；然而，在使用PT-Cy的单倍体移植中，KIR配体的不匹配对于临床结果的预测效果是不一致的[14],[16]–[17]。我们针对易发生移植物抗宿主病（Graft-versus-host disease，GVHD）的人群（接受母体和旁系供者的单倍体移植患者），在含有阿糖胞苷、白消安、Cy与标准剂量ATG/G-CSF北京方案基础上，移植后的第3、4天使用了低剂量（14.5 mg/kg）PT-Cy[18]–[19]。我们观察到，当受者具有供者提供的KIR配体（Ⅰ类），或者供者具有抑制性或激活性的KIR基因时，可以预防疾病复发[12],[20]–[23]。此外，受者-供者在HLA-B位点上的KIR配体匹配有助于重建NK细胞功能，从而预防移植后巨细胞病毒（Cytomegalovirus，CMV）的再激活[13]。然而，在基于ATG的单倍体移植环境中，很少有研究探讨PT-Cy的使用是否会影响供者和受者之间的KIR和HLA相互作用对临床结果的影响。

在供者选择方面，已有研究表明旁系相关供者（Collateral-related donors，CRD）和母体供者与急性移植物抗宿主病（Acute graft-versus-host disease，aGVHD）的风险增加相关[24]–[25]。因此，我们在母体或旁系供者的移植过程中增加了低剂量的PT-Cy[26]并对接受来自旁系相关供者以及接受或不接受PT-Cy的母体供者进行了受者的回顾性分析。首先，我们研究了在应用PT-Cy后，HLA-Bw4在临床结局和NK细胞教育中的预测作用是否发生变化。其次，我们探索了PT-Cy体内应用，HLA-Bw4与临床结局之间的关系是否受到影响，是否会对NK细胞教育产生影响。

## 病例与方法

一、病例资料

本研究包括两个队列。队列1是前瞻性招募的，其中包括8例AML或急性淋巴细胞白血病（Acute lymphoblastic leukemia, ALL）患者，他们在2020年至2021年间接受了来自母体或旁系亲属的单倍体移植和低剂量PT-Cy，以及24例在2017年至2021年期间未接受PT-Cy的倾向评分匹配（PSM）患者。队列2包括了2010年至2017年连续收治的278例血液系统恶性肿瘤患者，他们接受了来自母体或旁系亲属的单倍体移植。其中，在2015年至2017年，73例患者接受了低剂量PT-Cy；而在2010年至2014年，205例患者未接受PT-Cy。

二、预处理方案和GVHD预防

预处理方案包括阿糖胞苷、白消安、环磷酰胺、司莫司汀和兔ATG。GVHD预防方案包括环孢素A、霉酚酸酯和短期甲氨蝶呤。在预防感染和提供支持性护理方面，我们遵循了相关文献[27]–[28]中的经验和指导。

三、HLA分型与KIR配体分类

参照文献[29]报道的HLA分型细节，我们使用基于高分辨率DNA的方法来确定受者的HLA-B等位基因。在分析中考虑了KIR和HLA之间的相互作用，共有四个模型，包括：供者自身KIR（ds-KIR），在这种情况下，供者编码HLA配体Bw4，但受者不编码HLA配体Bw4；受者和供者都没有HLA-Bw4配体的非自身KIR（ns-KIR）；受者自身KIR（rs-KIR），在这种情况下，受者编码HLA配体Bw4，但供者不编码HLA配体Bw4；以及供者和受者都有HLA-Bw4配体的供者-受者自身KIR（d-rs-KIR）。供者中有HLA-Bw4配体的被称为d-rs+ds-KIR，没有HLA-Bw4配体的被称为rs+ns-KIR。

四、流式细胞术分析

对移植后30 d和90 d患者新鲜血液样本中的外周血单核细胞（PBMC）进行表面标记，包括CD3、CD56、CD335（NKp46）、CD337（NKp30）、CD57、DNAM1、NKG2C、NKG2A、NKG2D、KIR2DL1、KIR2DL2/L3、KIR3DL1、CD107a和IFN-γ。采用LSR Fortessa仪器进行数据采集，并使用FlowJo软件进行分析。使用单染色对照进行补偿，并进行数据归一化。在实验之前进行了配置验证和性能检查。

五、细胞毒性分析

细胞毒性实验使用来自患者的冷冻保存的PBMC样品。解冻后，PBMC经洗涤后转移到96孔板中，在含有胎牛血清和IL-2的培养基中培养10～14 h。细胞毒性测定使用CD107a和IFN-γ标记来评估不同NK细胞亚群对靶K562细胞的细胞毒作用。

六、统计学处理

在队列1中，我们使用PSM进行配对分析，包括以下变量进行匹配：诊断、患者年龄、供者年龄、患者性别、疾病风险指数和HLA等位基因匹配。队列2考虑了移植前的临床因素，包括患者年龄、移植前疾病风险和供者-受者KIR配体伙伴关系。移植前疾病风险分层包括低风险和高风险，高风险包括CR3或以上AML、CP2或以上慢性髓性白血病（CML）、CR3或以上ALL和骨髓增生异常综合征（MDS）-AML，低风险包括CR1/CR2期AML、CML-CP1、CR1/CR2 ALL或MDS-RCMD/RAEB（难治性贫血伴原始细胞过多/难治性血细胞减少伴多系病态造血）。队列2随访截至2019年6月，中位随访时间为684 d，总生存（Overall survival，OS）定义为从移植至死亡或最后一次随访的时间。无白血病生存（Leukemia-free survival，LFS）定义为从移植至白血病复发、死亡或最后一次随访的时间。移植相关死亡率（Transplantation-related mortality，TRM）指在从移植至因移植相关的并发症导致患者死亡的时间。研究使用国际骨髓移植协会（International Bone Marrow Transplantation Registry，IBMTR）/欧洲骨髓移植协会（European Group for Blood and Marrow Transplantation，EBMT）GVHD评分标准对aGVHD进行评估。使用Kaplan-Meier绘制生存曲线并估算生存概率，竞争风险分析用于计算GVHD、复发和TRM的累积发生率。采用SPSS 26.0、R 2.6.1和GraphPad Prism 8.0进行统计分析及数据可视化。

## 结果

一、患者特征

队列1的人口统计数据如下：接受母体或旁系亲属的PT-Cy单倍体移植的患者8例；来自非母体和无关供者的连续PSM匹配的未接受PT-Cy患者24例。在队列1中，接受和未接受PT-Cy组患者在年龄、性别、疾病类型、及KIR和HLA相互作用B位点方面差异均无统计学意义（表1）。

**表1:** 队列1的基本特征

分组特征	接受PT-Cy（8例）	未接受PT-Cy（24例）	*P*值
移植时年龄[岁，*M*（范围）]	31.5（18~40）	28（13~48）	0.380
男/女（例数）	4/4	15/9	0.413
诊断[例数（%）]			0.575
AML			
CR1	4（80.0）	13（81.2）	
≥CR2	1（20.0）	3（18.8）	
ALL			
CR1	3（100.0）	6（75.0）	
≥CR2	0（0.0）	2（25.0）	
KIR和HLA相互作用模型[例数（%）]			0.451
d-rs-KIR	6（75.00）	13（54.2）	
ds-KIR	1（12.5）	1（4.2）	
rs-KIR	0（0.0）	1（4.2）	
ns-KIR	1（12.5）	9（37.5）	

**注** PT-Cy：移植后环磷酰胺；AML：急性髓系白血病；CR：完全缓解；ALL：急性淋巴细胞白血病；KIR：杀伤细胞免疫球蛋白样受体；d-rs-KIR：供者和受者都有HLA-Bw4配体的供者-受者自身KIR；ds-KIR：供者自身KIR；rs-KIR：受者自身KIR；ns-KIR：受者和供者都没有HLA-Bw4配体的非自身KIR

队列2的人口统计数据如下：73例患者接受来自母体或旁系供者的单倍体HSCT联合PT-Cy方案预处理；205例接受来自母体或旁系供者的单倍体HSCT，未接受PT-Cy方案预处理。与接受PT-Cy组的患者相比，未接受PT-Cy组的患者更年轻［21（2～58）岁对27（6～40）岁，*P*<0.001］。接受PT-Cy组和未接受PT-Cy组患者其他临床特征差异无统计学意义（表2）。

**表2:** 队列2的基本特征

分组特征	接受PT-Cy （73例）	未接受PT-Cy （205例）	*P*值
移植时年龄[岁，*M*（范围）]	27（6~40）	21（2~58）	<0.001
男/女（例数）	44/29	114/91	0.490
诊断[例数（%）]			0.629
AML			
CR1	31（42.5）	65（31.7）	
≥CR2	3（4.1）	12（5.9）	
PR/NR	0	3（1.5）	
CML			
CP1	1（1.4）	1（0.5）	
CP2	3（4.1）	11（5.4）	
AP	0	1（0.5）	
BP	0	1（0.5）	
MDS			
RAEB/RCMD	7（9.6）	10（4.9）	
MDS-AML	1（1.4）	1（0.5）	
ALL			
CR1	25（34.2）	79（38.5）	
≥CR2	2（2.7）	15（7.3）	
NR	0	6（2.9）	
移植前疾病风险[例数（%）]			0.088
低风险	69（94.5）	179（87.3）	
高风险	4（5.5）	26（12.7）	
KIR和HLA相互作用模型[例数（%）]			0.961
d-rs-KIR	37（50.7）	97（47.3）	
ds-KIR	11（15.1）	31（15.1）	
rs-KIR	8（11.0）	24（11.7）	
ns-KIR	17（23.3）	53（25.9）	

**注** PT-Cy：移植后环磷酰胺；AML：急性髓系白血病；CR：完全缓解；PR：部分缓解；NR：未缓解；CML：慢性髓性白血病；CP：慢性期；AP：加速期；BP：急变期；MDS：骨髓增生异常综合征；RAEB：难治性贫血伴原始细胞过多；RCMD：难治性血细胞减少伴多系病态造血；ALL：急性淋巴细胞白血病；移植前疾病风险分层包括低风险和高风险，高风险包括CR3或以上AML期、CP2或以上CML期、CR3或更多ALL期和MDS-AML期患者，低风险包括CR1/CR2期AML、CML-CP1、CR1/CR2-期ALL或MDS-RCMD/RAEB。KIR：杀伤细胞免疫球蛋白样受体；d-rs-KIR：供者和受者都有HLA-Bw4配体的供者-受者自身KIR；ds-KIR：供者自身KIR；rs-KIR：受者自身KIR；ns-KIR：受者和供者都没有HLA-Bw4配体的非自身KIR

二、供者编码HLA-Bw4对NK细胞快速重建的影响

在队列1中，编码HLA配体Bw4（d-rs+ds-KIR）的供者组在第90天时，代表脱颗粒功能的CD107a^+^ NK细胞的表达高于没有HLA配体Bw4（rs+ns-KIR）的供者组（*P*＝0.046）（图1A）。此外，在整个队列1（*P*＝0.021）和未接受PT-Cy亚组（*P*＝0.048）中，在第30天时，d-rs+ds-KIR供者与rs+ns-KIR供者相比，NK细胞的比例增加（图1B）。

**图1:**
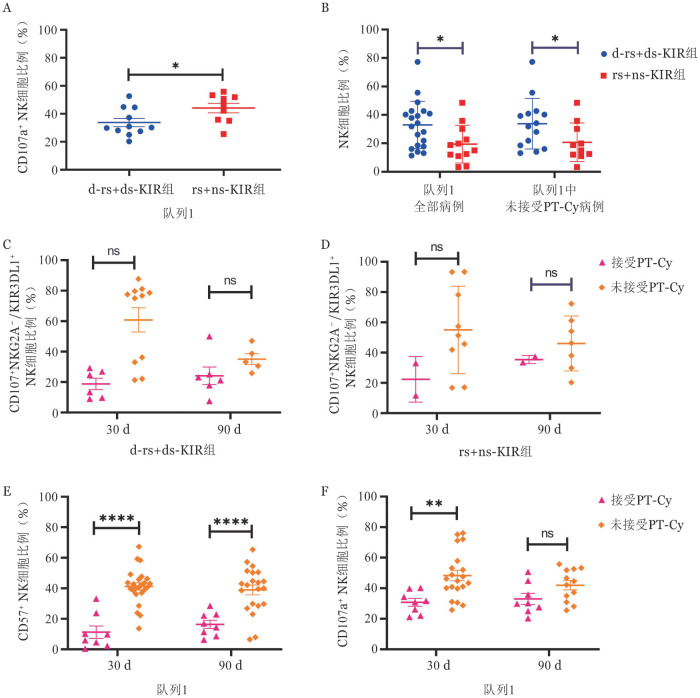
移植后环磷酰胺（PT-Cy）对供者HLA-Bw4预测NK教育效果的影响（*P<0.05, **P<0.01, ***P<0.01, ns示差异无统计学意义） **A** 移植后第90天d-rs+ds-KIR组、rs+ns-KIR组中CD107a^+^ NK细胞的比例；**B** 移植后第30天接受和不接受PT-Cy患者NK细胞的比例；**C** 移植后第30、90天，接受和不接受PT-Cy的病例中d-rs+ds-KIR组CD107^+^NKG2A^−^/KIR3DL1^+^ NK细胞的比例；**D** 移植后第30天和第90天，接受和不接受PT-Cy的病例中rs+ns-KIR组CD107^+^NKG2A^−^/KIR3DL1^+^ NK细胞的比例；**E** 移植后第30、90天，接受和不接受PT-Cy的病例中CD57^+^ NK细胞的比例；**F** 移植后第30、90天，接受和不接受PT-Cy的病例中CD107a^+^NK细胞的比例 **注** KIR：杀伤细胞免疫球蛋白样受体；d-rs-KIR：供者和受者都有HLA-Bw4配体的供者-受者自身KIR；ds-KIR：供者自身KIR；rs-KIR：受者自身KIR；ns-KIR：受者和供者都没有HLA-Bw4配体的非自身KIR

三、PT-Cy对NK细胞成熟和教育的影响

为了确定PT-Cy是否对NK细胞的教育产生影响，我们比较了在接受和未接受PT-Cy的病例之间进行了NKG2A^−^/KIR3DL1^+^ NK细胞。在第30天和第90天，在d-rs+ds-KIR供者或rs+ns-KIR供者中，接受PT-Cy和未接受PT-Cy的病例之间的CD107a^+^ NKG2A^−^/KIR3DL1^+^ NK细胞比例差异无统计学意义（图1C、D）。然而，在第30天和第90天，接受PT-Cy的病例中，CD57^+^ NK细胞在NK细胞中的比例低于未接受PT-Cy的病例（*P*值均<0.001）（图1E）。CD107a^+^ NK细胞的比例（*P*＝0.002）在接受PT-Cy的患者中较第30天减少，但在第90天时与未接受PT-Cy的患者差异无统计学意义（图1F）。

四、不同HLA-Bw4分类对接受PT-Cy治疗的患者生存的影响

我们对队列2中接受PT-Cy治疗的患者中供受者HLA-Bw4配体对临床结果的影响进行了分析。在接受PT-Cy治疗的患者中，根据供者的KIR基因型进行分组，包括ds-KIR组（11例）、rs-KIR组（8例）、ns-KIR组（17例）和d-rs KIR组（37例），四组3年LFS率差异无统计学意义［（81.8±11.6）％、（60.0±18.2）％、（65.5±13.4）％、（71.4±9.3）％，*P*＝0.671］，3年OS率（*P*＝0.381）和累积复发率（*P*＝0.668）差异均无统计学意义。

在考虑死亡原因时，我们注意到该队列中的所有死亡事件与严重感染有关。这些感染包括肠道感染、软组织感染、腹部感染、肺部多重感染、巨细胞病毒肺炎、EB病毒相关的移植后淋巴增殖性疾病（Post-transplant lymphoproliferative disorders，PTLD）以及乙型肝炎复发引起的肝功能衰竭。进一步分析结果显示，在考虑TRM时，我们观察到接受来自rs-KIR供者的患者TRM较高［（40.0±4.0）％］，而接受ns-KIR［（22.7±1.7）％］、ds-KIR（0％）和d-rs KIR［（7.3±0.3）％］供者的患者TRM较低（*P*＝0.033）（图2A）。d-rs+ds-KIR组与rs+ns-KIR组相比，前者显示出较低的TRM率［（5.8±0.2）％对（29.0±1.2）％，*P*＝0.009］（图2B）。

**图2:**
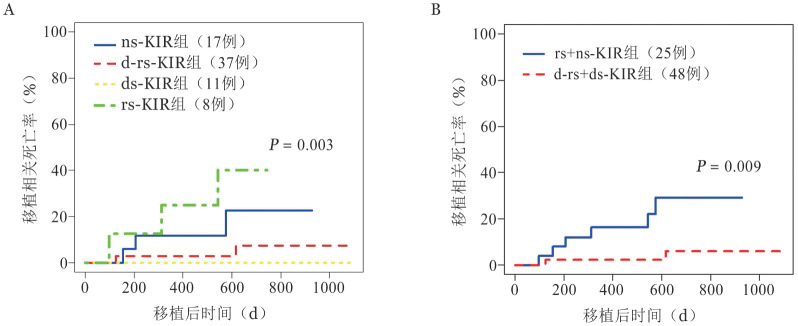
HLA-Bw4供者四分类（A）和两分类（B）对PT-Cy患者的移植相关死亡率的影响 **注** KIR：杀伤细胞免疫球蛋白样受体；d-rs-KIR：供者和受者都有HLA-Bw4配体的供者-受者自身KIR；ds-KIR：供者自身KIR；rs-KIR：受者自身KIR；ns-KIR：受者和供者都没有HLA-Bw4配体的非自身KIR

我们还评估了编码HLA-Bw4配体的供者对aGVHD的影响。ds+d-rs-KIR组与rs+ns-KIR组相比，Ⅱ～Ⅳ级［（22.92±0.38）％对（16.00±0.56）％，*P*＝0.407］和Ⅲ～Ⅳ级［（2.08±0.04）％对（8.00±0.31）％，*P*＝0.237］aGVHD的发生率差异均无统计学意义。

五、编码HLA-Bw4的供者对未接受PT-Cy患者的生存的影响

在未接受PT-Cy的队列2中，根据HLA-B位点上不同的供受者配对关系，rs-KIR组5年LFS和OS率分别为（39.5±11.0）％、（46.1±10.6）％，ds-KIR组分别为（57.0±9.0）％、（59.8±9.1）％，d-rs KIR组分别为（68.0±4.8）％、（71.9±4.7）％，ns-KIR组分别为（63.9±6.6）％和（65.8±6.6）％（*P*值分别为0.070、0.058）。在上述指标方面，各组之间的差异无统计学意义。

rs-KIR、ds-KIR、d-rs-KIR及ns-KIR组的TRM分别为（40.0±1.2）％、（26.2±0.7）％、（12.6±0.1）％和（22.9±0.3）％（*P*＝0.017）（图3A）；感染相关TRM分别为（35.7±1.1）％、（23.0±0.6）％、（10.4±0.1）％和（21.0±0.3）％（*P*＝0.025）（图3B）。d-rs+ds-KIR组与rs+ns-KIR组相比具有较低的TRM［（15.89±0.11）％对（28.00±0.27）％，*P*＝0.044］（图3C）以及感染相关的TRM［（13.46±0.09）％对（25.37±0.26）％，*P*＝0.040］（图3D）。

**图3:**
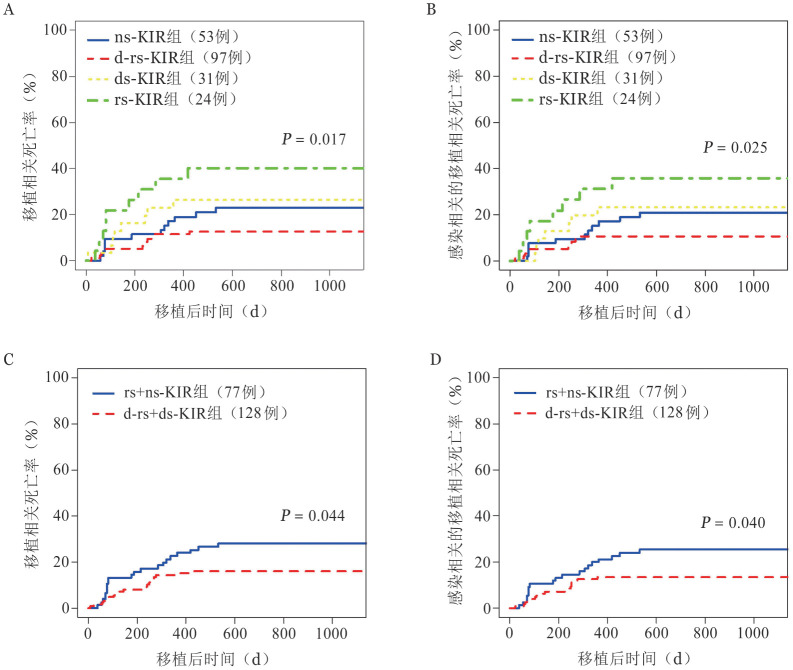
HLA-Bw4供者四分类（A、B）与二分类（C、D）对未接受PT-Cy病例中的移植相关死亡率（A、C）及感染相关移植相关死亡率（B、D）的影响 **注** KIR：杀伤细胞免疫球蛋白样受体；d-rs-KIR：供者和受者都有HLA-Bw4配体的供者-受者自身KIR；ds-KIR：供者自身KIR；rs-KIR：受者自身KIR；ns-KIR：受者和供者都没有HLA-Bw4配体的非自身KIR

ds+d-rs-KIR组与rs+ns-KIR组相比，Ⅱ～Ⅳ级［（36.22±0.18）％对（42.86±0.32）％，*P*＝0.283］和Ⅲ～Ⅳ级［（4.69±0.04）％对（11.69±0.14）％，*P*＝0.064］aGVHD的发生率差异无统计学意义。

六、队列2的多因素分析

在队列2中，我们进行了多因素分析，进一步展示了供受者HLA-Bw4模型对TRM的影响。纳入的指标包括患者年龄、疾病类型、移植前疾病风险、供受者配体模型和移植类型（是否接受PT-Cy）。结果显示，rs+ns-KIR组合［*HR*＝2.229（95％*CI* 1.269～3.916），*P*＝0.005］和移植前高风险状态［*HR*＝2.180（95％*CI* 1.017～4.670），*P*＝0.045］与较高的TRM相关。此外，高风险疾病状态和淋巴系统恶性肿瘤与较高的复发率（*P*<0.001，*P*＝0.001）和较低的LFS率（*P*<0.001，*P*＝0.014）有关。高风险疾病状态与较低的OS率相关（*P*<0.001）。

## 讨论

过去的研究结果对于不同供者类型和预处理方法在单倍体移植中NK细胞同种异体反应的作用存在不一致性[7],[23],[31]–[36]。本研究的创新之处在于关注母体或旁系供者的单倍体移植，并通过对照研究比较接受和未接受PT-Cy的情况，探讨了PT-Cy对供受者KIR配体在临床结果上的影响。

由于NK细胞在感染性病原体的先天免疫反应中起到关键作用，rs-KIR或ns-KIR可能会干扰母体或旁系供者提供的有效NK细胞重建和异体反应在特定受者环境中的作用。我们假设具有编码HLA配体Bw4的供者能够更迅速地重建NK细胞，并在控制各种感染方面具有更强的能力，从而在这种情况下有助于降低与感染相关的TRM。我们发现HLA-Bw4阳性供者具有更高的细胞毒性和更高的NK细胞重建比例。然而，在编码HLA配体Bw4（d-rs+ds）的供者和不编码HLA配体Bw4（rs+ns）的供者之间，并没有观察到NKG2A^−^/KIR3DL1^+^ NK细胞教育方面的差异。以前的研究发现KIR3DL1和HLA-BW4的组合对NK细胞教育具有积极影响，表现为KIR3DL1^+^ NK细胞具有更高的敏感性和比例[11],[37]–[38]。然而，在我们的队列中只有1例患者的疾病风险较高（CR≥3），81.25％的患者达到CR1，这可能是免疫重建良好的原因。

既往研究表明，在单倍体移植后应用PT-Cy会对NK细胞重建产生不良影响：在移植后的早期阶段，扩增的不成熟CD56^bright^CD16^−^NK细胞数量明显减少。在移植后30 d，不成熟的NK细胞亚群表达高水平的NKG2A，较低水平的CD57和KIR。然而，在移植后90 d，这些细胞数量部分恢复[15]。PT-Cy的作用机制是选择性清除正在分裂的细胞，通过减少正在增殖的NK细胞，导致不成熟的NK细胞（CD62L^+^NKG2A^+^KIR^−^）大量存在[14]。与以前的研究一致，我们的研究也发现，在移植后早期，PT-Cy抑制了NK细胞的成熟。然而，我们的研究结果显示，PT-Cy的应用并没有改变编码HLA-Bw4的供者组中NKG2A^−^/KIR3DL1^+^ NK细胞教育。这意味着PT-Cy对于这些特定的NK细胞亚群的发育没有产生显著影响。

尽管我们的研究结果和既往研究都显示PT-Cy抑制NK细胞成熟[14]–[15]，但我们的回顾性队列研究表明，PT-Cy对于编码HLA-Bw4的供者在临床结果上的影响并没有改变。我们观察到，不论是否使用PT-Cy，编码HLA-Bw4的供者在降低TRM和感染相关的TRM方面具有优势。其中一个原因是NK细胞的教育可能在其抗感染功能中发挥更重要的作用，并且PT-Cy在体内应用并不影响NK细胞的教育。此外，我们的研究结果以及最近的研究都表明，PT-Cy在移植后的早期阶段的抑制作用是有限的，因此对于长期的TRM没有影响。

在我们的研究中，我们没有观察到不同供受者KIR配体模型对于Bw4在白血病复发或GVHD方面的影响。然而，纽约的一项研究发现，在进行HLA相合的AML患者的HLA相合HSCT时，具有强抑制性KIR3DL1/HLA-Bw4组合的患者预示着较高的复发率。此外，原始KIR3DL1^+^ NK细胞对于HLA-Bw4亚型的抑制能力存在差异，这限制了它们对白血病细胞的靶向作用能力[9]。我们之前的研究发现，对于具有抑制性KIR受体的受体KIR配体的供者，在单倍体移植中，有助于NK细胞受到许可，并提供了最大的T细胞重建保护效果[12],[14],[16],[21]。出现不同研究结果可能是由于以下因素所致：①不同的疾病类型；②不同的供受者关系；③不同的移植类型和预处理方案；④没有考虑供者KIR3DL1等位基因的影响。这些因素可能通过影响NK细胞的重建和教育而导致结果的多样性。

我们的研究受到一些限制，包括回顾性分析和部分患者随访时间相对较短的限制。此外，在队列2中，未接受PT-Cy处理的患者并非全部来自母体或旁系供者。尽管存在这些限制，本研究是在以ATG为基础的单倍型相合移植模式中首次提供了有关PT-Cy在体内应用对NK细胞作用的数据。

综上所述，我们的研究结果表明，编码HLA-Bw4的供者有助于促进NK细胞的重建和功能，而这种作用在体内应用PT-Cy时并不受影响。相反，不编码HLA-Bw4的供者与更高的严重感染风险相关，从而增加了治疗相关死亡的风险。我们需要进行更多的前瞻性临床研究和功能实验研究来帮助更深入地理解HLA-Bw4的作用以及PT-Cy在NK细胞功能上的影响。

## References

[1] Chang YJ, Zhao XY, Huang XJ (2008). Effects of the NK cell recovery on outcomes of unmanipulated haploidentical blood and marrow transplantation for patients with hematologic malignancies[J]. Biol Blood Marrow Transplant.

[2] Zhao XY, Huang XJ, Liu KY (2007). Reconstitution of natural killer cell receptor repertoires after unmanipulated HLA-mismatched/haploidentical blood and marrow transplantation: analyses of CD94:NKG2A and killer immunoglobulin-like receptor expression and their associations with clinical outcome[J]. Biol Blood Marrow Transplant.

[3] Pfeiffer MM, Feuchtinger T, Teltschik HM (2010). Reconstitution of natural killer cell receptors influences natural killer activity and relapse rate after haploidentical transplantation of T- and B-cell depleted grafts in children[J]. Haematologica.

[4] Chang YJ, Zhao XY, Huang XJ (2018). Strategies for Enhancing and Preserving Anti-leukemia Effects Without Aggravating Graft-Versus-Host Disease[J]. Front Immunol.

[5] Shimoni A, Vago L, Bernardi M (2017). Missing HLA C group 1 ligand in patients with AML and MDS is associated with reduced risk of relapse and better survival after allogeneic stem cell transplantation with fludarabine and treosulfan reduced toxicity conditioning[J]. Am J Hematol.

[6] Björklund AT, Schaffer M, Fauriat C (2010). NK cells expressing inhibitory KIR for non-self-ligands remain tolerant in HLA-matched sibling stem cell transplantation[J]. Blood.

[7] Vago L, Forno B, Sormani MP (2008). Temporal, quantitative, and functional characteristics of single-KIR-positive alloreactive natural killer cell recovery account for impaired graft-versus-leukemia activity after haploidentical hematopoietic stem cell transplantation[J]. Blood.

[8] Martin MP, Qi Y, Gao X (2007). Innate partnership of HLA-B and KIR3DL1 subtypes against HIV-1[J]. Nat Genet.

[9] Boudreau JE, Giglio F, Gooley TA (2017). KIR3DL1/HLA-B Subtypes Govern Acute Myelogenous Leukemia Relapse After Hematopoietic Cell Transplantation[J]. J Clin Oncol.

[10] Hallner A, Bernson E, Hussein BA (2019). The HLA-B-21 dimorphism impacts on NK cell education and clinical outcome of immunotherapy in acute myeloid leukemia[J]. Blood.

[11] Zhao XY, Yu XX, Xu ZL (2019). Donor and host coexpressing KIR ligands promote NK education after allogeneic hematopoietic stem cell transplantation[J]. Blood Adv.

[12] Zhao XY, Chang YJ, Zhao XS (2015). Recipient expression of ligands for donor inhibitory KIRs enhances NK-cell function to control leukemic relapse after haploidentical transplantation[J]. Eur J Immunol.

[13] Zhao XY, Luo XY, Yu XX (2017). Recipient-donor KIR ligand matching prevents CMV reactivation post-haploidentical T cell-replete transplantation[J]. Br J Haematol.

[14] Russo A, Oliveira G, Berglund S (2018). NK cell recovery after haploidentical HSCT with posttransplant cyclophosphamide: dynamics and clinical implications[J]. Blood.

[15] Rambaldi B, Kim HT, Reynolds C (2021). Impaired T- and NK-cell reconstitution after haploidentical HCT with posttransplant cyclophosphamide[J]. Blood Adv.

[16] Willem C, Makanga DR, Guillaume T (2019). Impact of KIR/HLA Incompatibilities on NK Cell Reconstitution and Clinical Outcome after T Cell-Replete Haploidentical Hematopoietic Stem Cell Transplantation with Posttransplant Cyclophosphamide[J]. J Immunol.

[17] Shimoni A, Labopin M, Lorentino F (2019). Killer cell immunoglobulin-like receptor ligand mismatching and outcome after haploidentical transplantation with post-transplant cyclophosphamide[J]. Leukemia.

[18] Wang Y, Wu DP, Liu QF (2019). Low-dose post-transplant cyclophosphamide and anti-thymocyte globulin as an effective strategy for GVHD prevention in haploidentical patients[J]. J Hematol Oncol.

[19] Wang Y, Chang YJ, Chen L (2017). Low-dose post-transplant cyclophosphamide can mitigate GVHD and enhance the G-CSF/ATG induced GVHD protective activity and improve haploidentical transplant outcomes[J]. Oncoimmunology.

[20] Wang Y, Liu QF, Lin R (2021). Optimizing antithymocyte globulin dosing in haploidentical hematopoietic cell transplantation: long-term follow-up of a multicenter, randomized controlled trial[J]. Sci Bull (Beijing).

[21] Zhao XY, Huang XJ, Liu KY (2007). Prognosis after unmanipulated HLA-haploidentical blood and marrow transplantation is correlated to the numbers of KIR ligands in recipients[J]. Eur J Haematol.

[22] Huang XJ, Zhao XY, Liu DH (2007). Deleterious effects of KIR ligand incompatibility on clinical outcomes in haploidentical hematopoietic stem cell transplantation without in vitro T-cell depletion[J]. Leukemia.

[23] Zhao XY, Chang YJ, Xu LP (2014). HLA and KIR genotyping correlates with relapse after T-cell-replete haploidentical transplantation in chronic myeloid leukaemia patients[J]. Br J Cancer.

[24] Mo XD, Zhang YY, Zhang XH (2018). The role of collateral related donors in haploidentical hematopoietic stem cell transplantation[J]. Sci Bull (Beijing).

[25] Wang Y, Chang YJ, Xu LP (2014). Who is the best donor for a related HLA haplotype-mismatched transplant?[J]. Blood.

[26] Wang Y, Chang YJ, Chen L (2017). Low-dose post-transplant cyclophosphamide can mitigate GVHD and enhance the G-CSF/ATG induced GVHD protective activity and improve haploidentical transplant outcomes[J]. Oncoimmunology.

[27] Wang Y, Liu DH, Liu KY (2013). Long-term follow-up of haploidentical hematopoietic stem cell transplantation without in vitro T cell depletion for the treatment of leukemia: nine years of experience at a single center[J]. Cancer.

[28] Liu D, Huang X, Liu K (2008). Haploidentical hematopoietic stem cell transplantation without in vitro T cell depletion for treatment of hematological malignancies in children[J]. Biol Blood Marrow Transplant.

[29] Lu DP, Dong L, Wu T (2006). Conditioning including antithymocyte globulin followed by unmanipulated HLA-mismatched/haploidentical blood and marrow transplantation can achieve comparable outcomes with HLA-identical sibling transplantation[J]. Blood.

[30] Armand P, Kim HT, Logan BR (2014). Validation and refinement of the Disease Risk Index for allogeneic stem cell transplantation[J]. Blood.

[31] Ruggeri L, Mancusi A, Capanni M (2007). Donor natural killer cell allorecognition of missing self in haploidentical hematopoietic transplantation for acute myeloid leukemia: challenging its predictive value[J]. Blood.

[32] Beelen DW, Ottinger HD, Ferencik S (2005). Genotypic inhibitory killer immunoglobulin-like receptor ligand incompatibility enhances the long-term antileukemic effect of unmodified allogeneic hematopoietic stem cell transplantation in patients with myeloid leukemias[J]. Blood.

[33] Giebel S, Locatelli F, Lamparelli T (2003). Survival advantage with KIR ligand incompatibility in hematopoietic stem cell transplantation from unrelated donors[J]. Blood.

[34] Kröger N, Shaw B, Iacobelli S (2005). Comparison between antithymocyte globulin and alemtuzumab and the possible impact of KIR-ligand mismatch after dose-reduced conditioning and unrelated stem cell transplantation in patients with multiple myeloma[J]. Br J Haematol.

[35] Symons HJ, Leffell MS, Rossiter ND (2010). Improved survival with inhibitory killer immunoglobulin receptor (KIR) gene mismatches and KIR haplotype B donors after nonmyeloablative, HLA-haploidentical bone marrow transplantation[J]. Biol Blood Marrow Transplant.

[36] Davies SM, Ruggieri L, DeFor T (2002). Evaluation of KIR ligand incompatibility in mismatched unrelated donor hematopoietic transplants. Killer immunoglobulin-like receptor[J]. Blood.

[37] Boudreau JE, Mulrooney TJ, Le Luduec JB (2016). KIR3DL1 and HLA-B Density and Binding Calibrate NK Education and Response to HIV[J]. J Immunol.

[38] Lisovsky I, Kant S, Tremblay-McLean A (2019). Differential contribution of education through KIR2DL1, KIR2DL3, and KIR3DL1 to antibody-dependent (AD) NK cell activation and ADCC[J]. J Leukoc Biol.

